# New Medical Journal

**Published:** 1859-11

**Authors:** 


New Medical Journal.—We have received the first nine numbers of the *Journal de Progrès des Sciences Médicales et de L'Hydrothérapie Rationelle*, edited by M. Louis Fleury, with the assistance of an able corps of collaborators, among whom we notice such names as Malgaigne, Nélaton, Gosselin, Jobert de Lamballe, Piorry and others, a sufficient guaranty that the periodical will be sustained in a masterly manner. Dr. Fleury was formerly connected with *Le Progrès*, a journal which existed about eighteen months, during which time he made many bold and meritorious attacks upon charlatanism, for which he has had to defend four actions at law, two being decided against him. We gladly place his journal on our exchange list.

